# Orthosteric and Allosteric Ligands of Nicotinic Acetylcholine Receptors for Smoking Cessation

**DOI:** 10.3389/fnmol.2015.00071

**Published:** 2015-11-25

**Authors:** Tasnim S. Mohamed, Selwyn S. Jayakar, Ayman K. Hamouda

**Affiliations:** ^1^Department of Pharmaceutical Sciences, College of Pharmacy, Texas A&M Health Sciences CenterKingsville, TX, USA; ^2^Department of Neurobiology, Harvard Medical SchoolBoston, MA, USA; ^3^Department of Neuroscience and Experimental Therapeutics, College of Medicine, Texas A&M Health Sciences CenterBryan, TX, USA

**Keywords:** smoking cessation, nicotine addiction, nicotinic acetylcholine receptor (nAChR), positive allosteric modulator, drug development

## Abstract

Nicotine addiction, the result of tobacco use, leads to over six million premature deaths world-wide per year, a number that is expected to increase by a third within the next two decades. While more than half of smokers want and attempt to quit, only a small percentage of smokers are able to quit without pharmacological interventions. Therefore, over the past decades, researchers in academia and the pharmaceutical industry have focused their attention on the development of more effective smoking cessation therapies, which is now a growing 1.9 billion dollar market. Because the role of neuronal nicotinic acetylcholine receptors (nAChR) in nicotine addiction is well established, nAChR based therapeutics remain the leading strategy for smoking cessation. However, the development of neuronal nAChR drugs that are selective for a nAChR subpopulation is challenging, and only few neuronal nAChR drugs are clinically available. Among the many neuronal nAChR subtypes that have been identified in the brain, the α4β2 subtype is the most abundant and plays a critical role in nicotine addiction. Here, we review the role of neuronal nAChRs, especially the α4β2 subtype, in the development and treatment of nicotine addiction. We also compare available smoking cessation medications and other nAChR orthosteric and allosteric ligands that have been developed with emphasis on the difficulties faced in the development of clinically useful compounds with high nAChR subtype selectivity.

## Introduction

Tobacco smoking is considered the leading preventable cause of disease, disability, and death worldwide (WHO report on the global tobacco epidemic 2011). According to the recent report of the Center for Disease and Control and the 32nd tobacco-related Surgeon General’s Report published in 2014, smoking remains the single largest cause of preventable disease and death in USA (Alberg et al., 2014). There are about 45 million smokers in the USA leading to more than 400,000 premature deaths and over $300 billion in health care expenses and lost productivity each year. Smoking prevalence in USA has declined in the last 50 years, in part, because of public awareness and governmental regulation that control tobacco use. However, smoking prevalence among adults with serious mental illness has increased 53% (Evins et al., 2015). In addition, an estimated 16 million Americans suffer from tobacco-related illnesses and 126 million Americans are exposed to secondhand smoking. Lung cancer is the primary contributor to tobacco-related illnesses; however, tobacco smoking also increases the risk of coronary heart disease, stroke and chronic obstructive pulmonary disease (Surgeon General’s Report, 2014).

Tobacco smoke contains hundreds of chemicals, many of which are known carcinogens; however, nicotine is the only component with known addictive properties (Wonnacott et al., 2005). The ventral tegmental area (VTA) dopaminergic (DA) neurons, which send projections to the nucleus accumbens (NAc) are highly implicated in rewarding and aversive effects of nicotine (Klink et al., 2001; Laviolette and van der Kooy, 2004; Kalamida et al., 2007; Changeux, 2010). Nicotine modulates the firing rate of VTA DA neurons and the levels of dopamine in the NAc by acting directly on nicotinic acetylcholine receptors (nAChRs) expressed on these neurons (Picciotto et al., 1995, 1998). Nicotine also indirectly modulates VTA DA neurons firing rate through binding to nAChRs on GABAergic and glutamatergic neurons projecting to the VTA (Mansvelder et al., 2002). In the absence of nicotine, VTA DA neurons are characterized by spontaneous single-spike firing (Gracem and Bunney, 1984). However, in the presence of nicotine, burst firing of VTA DA neurons can be observed which subsequently increase in the level of extracellular dopamine in the NAc (Nisell et al., 1994; Erhardt et al., 2002). With prolonged use of nicotine products, dopaminergic reward pathways become “sensitized” to nicotine and nicotine dependance becomes evident by the presence of withdrawal symptoms such as anxiety, irritability, and stress (Dome et al., 2010). Once nicotine dependance is developed, it requires continuous nicotine reinforcement and becomes difficult to quit smoking.

**Figure 1:**
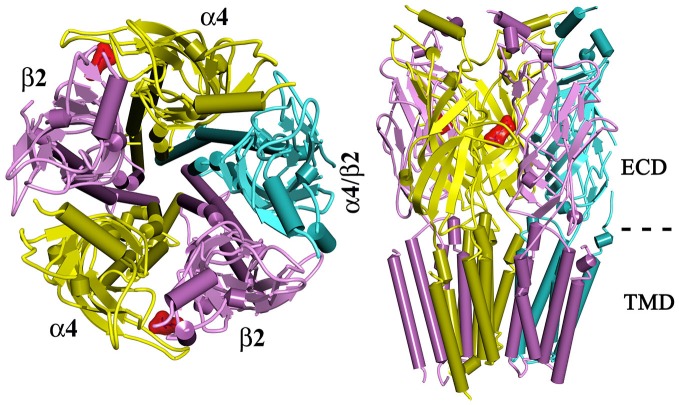
**A top view (*left*) and a side view (*right*) of a homology model of α4β2 nAChR.** The α4β2 nAChR contains two α4 subunits, two β2 subunits, and a fifth subunit that can be α4, β2, or other nAChR subunit. Two orthosteric (agonist-binding) sites are located at the extracellular domain of α4 and β2 subunit (denoted by a docked ligand in red). ECD, extracellular domain; TMD, Transmembrane domain.

Neuronal-type nAChRs are pentameric ligand gated ion channels that mediate the action of the endogenous neurotransmitter acetylcholine. Twelve homologous neuronal nAChR subunits, nine α subunits (α2–α10) and three β subunits (β2–β4), form functional nAChRs that are either homopentamers (e.g., α7 nAChR) or heteropentamers (e.g., α4β2; Figure 1; Gotti and Clementi, 2004; Kalamida et al., 2007; Hurst et al., 2013). Each nAChR contains at least two orthosteric (agonist-binding, canonical) sites at the extracellular interface between an α subunit and a β subunit (e.g., α4:β2 interface) or between two α subunits (e.g., α7:α7 interface; Jensen et al., 2005). In addition to the orthosteric sites, allosteric binding sites have been identified within the extracellular and transmembrane domains of nAChRs (reviewed in Hamouda et al., 2014).

The α4β2 nAChR is the most abundant and diffuse neuronal nAChR subtype. It has a high affinity for nicotine (*Ki*, ~1 nM) representing >90% of high-affinity nicotine binding sites in the brain and is a primary nAChRs subtype affected by nicotine blood levels achieved during smoking (Benwell et al., 1988; Flores et al., 1992; Rose et al., 1999; Sabey et al., 1999). Chronic exposure of α4β2 nAChR to nicotine causes receptor desensitization and upregulation, which are thought to play key roles in nicotine reinforcement leading to addiction (Reus et al., 2007). Both α4 knock-out (KO) mice and β2 KO mice lack the majority of high-affinity nicotine binding sites (Picciotto et al., 1995; Marubio et al., 1999). Alpha-4 KO mice display reduced antinociceptive effects of nicotine and lack the high-affinity nicotine-induced response in thalamic neurons (Marubio et al., 1999). Beta-2 KO mice do not self-administer nicotine, lack nicotine-induced improvement of performance in passive avoidance tests and exhibit no increase in VTA currents and striatal dopamine levels in response to nicotine (Picciotto et al., 1995, 1998). Targeted re-expression of the β2 subunit in the VTA of mice lacking β2 nAChR subunit (β2 KO) was sufficient to reestablish sensitivity to nicotine (Maskos et al., 2005). These results establish that α4β2 nAChRs in neurons, originating in the VTA, play a crucial role in nicotine addiction.

In addition to α4 and β2, other nAChR subunits especially α6 and α5 have been shown to play a role in nicotine addiction, albeit as part of a β2-containing receptors (Changeux, 2010; Brunzell et al., 2014). Alpha 6-containing nAChRs (e.g., α6β2 and α4α6β2) are expressed in the VTA DA neuron cell bodies and thought to mediate 30–80% of nicotine-induced striatal and NAc dopamine release (Drenan et al., 2008; Exley et al., 2008; Zhao-Shea et al., 2011). An α6 KO mouse exhibits reduced burst firing of DA neurons and decreased nicotine self-administration (Changeux, 2010) suggesting a possible role in mediating changes in burst firing in DA neurons of the VTA. The α5 nAChR subunit is expressed in the reward pathway and forms a functional receptor with α4 and β2 subunits replacing a β2 subunit (Changeux, 2010; Jin et al., 2014). Alpha 5-containing nAChRs have been shown to regulate dopamine transmission in the dorsal striatum and to determine the aversive response to nicotine (Frahm et al., 2011; Exley et al., 2012). Furthermore, an α5 subunit variant containing a single aspartic acid to asparagine substitution (α5D398N) is associated with an increased risk of developing nicotine dependance (Bierut et al., 2008). Because α6- and α5 nAChR subunits have limited brain distribution compared with other nAChR subunits, targeting α6- and α5-containing receptors is a promising strategy to develop tobacco cessation therapeutics with lower prevalence of adverse effects (Gotti et al., 2010).

## Nicotine Addiction Pharmacotherapies

**Figure 2:**
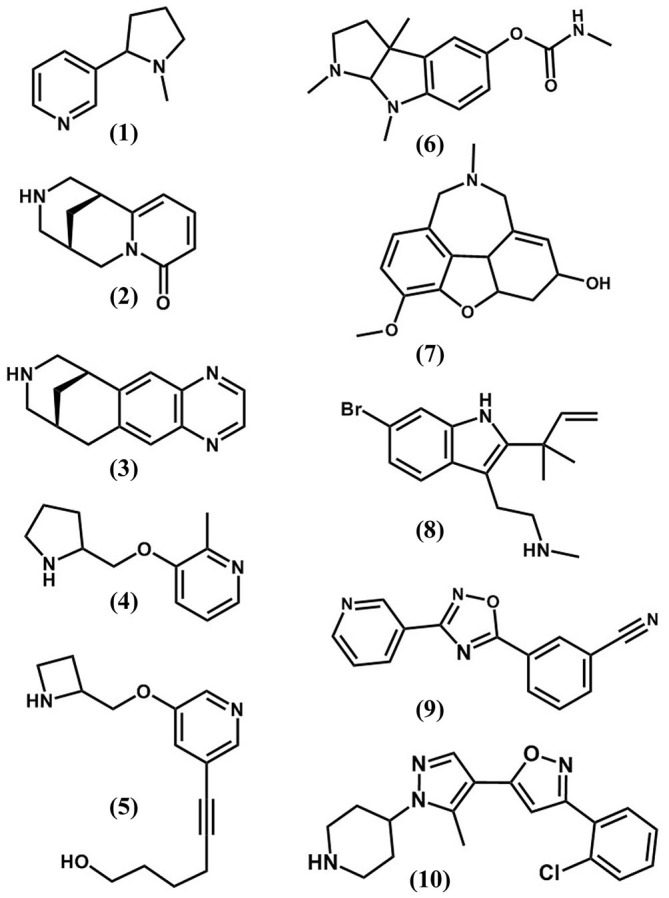
**The chemical structures of nAChR orthosteric (1–5) and allosteric (6–10) ligands.** (1) nicotine; (2), cytisine; (3), varenicline; (4), ABT-089; (5), sazetidine; (6), physostigmine; (7), galanthamine; (8), dFBr; (9), NS9283; (10), CMPI.

Tobacco addiction is a chronic condition that in most cases requires a combination of behavioral and pharmacological therapies. The majority of smokers want (69%) and attempt (52%) to quit, however, only a small percentage of smokers succeed even with the help of currently available interventions (Gonzales et al., 2006). Because nAChRs play a central role in the neuronal mechanism leading to nicotine addiction, they are considered a major target for the development of therapeutic strategies for smoking cessation aids. Current FDA-approved therapeutic interventions for smoking cessation include nicotine, bupropion and varenicline (Figure 2). Other smoking cessation medications include cytisine as well as few second-line medications such as clonidine and nortriptyline (Lerman et al., 2007; Syad and Chaudhari, 2013).

### Nicotine Replacement Treatments

Nicotine replacement treatments (NRTs) are FDA approved and the most prevalent pharmacological interventions for smoking cessation. NRT products include transdermal patches, chewing gums, nasal sprays, inhalers, orally dissolvable films, lozenges and low nicotine cigarettes. NRTs increase the chances of quitting when compared with placebo or a lack of treatment. However, over 90% of over-the-counter NRT users relapse within six months (Stead et al., 2008).

### Cytisine (Tabex^®^ (EU); Sopharma/Extab)

Cytisine is the world’s oldest smoking cessation aid (Prochaska et al., 2013). It is an α4β2 nAChR partial agonist that is widely used in Europe for smoking cessation but not approved by the FDA for use in the United States. Cytisine has clinical efficacy (end-of treatment odd ratio of 1.93–3.4) that is comparable to varenicline and NRT but associated with higher frequency of adverse effects (Etter, 2006; West et al., 2011; Leaviss et al., 2014). When combined with behavioral support, cytisine was superior to NRT as a smoking cessation aid (Walker et al., 2014).

### Varenicline (Chantix^®^ (USA), Champix^®^ (EU); Pfizer)

Varenicline is a cytisine derivative that acts as a partial agonist at α4β2 nAChR and a full agonist at α7 nAChR, but its efficacy as a smoking cessation aid correlates with its effect on α4β2 nAChR (Mihalak et al., 2006; Rollema et al., 2007). Varenicline displays ~30–60% of the *in vivo* efficacy of nicotine and blocks nicotine response both *in vivo* and *in vitro* (Coe et al., 2005a). In randomized controlled trials, smokers receiving varenicline initially have higher abstinence rates than those receiving placebo or any other smoking cessation treatments (end-of-treatment odds ratio of 1.7–4.9; Tonstad et al., 2006; Reus et al., 2007; Nides et al., 2008). The abstinence rate was higher with longer varenicline treatment (24 vs. 6 weeks; Lee et al., 2008) but the superiority of varenicline compared with other treatments became less significant at longer end points (Gonzales et al., 2006). Along with cognitive behavioral therapy, varenicline improved prolonged abstinence rates for smokers with serious mental illnesses (Evins et al., 2014). Varenicline’s association with behavioral side effects, including abnormal dreams, depression, and suicidal thoughts, led to the inclusion of boxed warnings for serious neuropsychiatric risks.

### Bupropion (Wellbutrin^®^/Zyban^®^; GlaxoSmithKline)

Bupropion was used first as an antidepressant acting through the modulation of monoamine neurotransmitters and then it was approved by the FDA as the first non-nicotine medication for use in smoking cessation (Lerman et al., 2007). Bupropion inhibits α4β2 and α7 nAChRs, inhibits nicotine-induced dopamine release and attenuates nicotine self-administration (Slemmer et al., 2000; Dwoskin et al., 2006). Since adults with depression are more likely to smoke, the antidepressant effect of bupropion is very advantageous in decreasing stress and negative mood (dysphoric-like) state associated with nicotine withdrawal. Diminishing the dysphoric-like state associated with nicotine withdrawal is believed to prevent relapse to smoking and has been shown with other smoking cessation agents such as varenicline and cytisine (Igari et al., 2014). As monotherapy, bupropion has a lower abstinence rates compared with varenicline (Nides et al., 2008). However, in combination with varenicline, bupropion treatment has been shown to increase rates of long-term abstinence from smoking but not 7-day point prevalence at 12 and 26 weeks (Ebbert et al., 2014).

## Investigational nAChR Orthosteric Ligands for Smoking Cessation

Current tobacco dependance treatments have a 12-month abstinence success rate of 22% at best (Gonzales et al., 2006); the relapse rate within 1 year following the discontinuation of smoking cessation therapy is high (Tonstad et al., 2006). Therefore, a critical need for more effective smoking cessation aids still exists. Several nAChR ligands (partial agonists, antagonists, or desensitizers; Figure 2) are currently under clinical trials for use as smoking cessation therapies or have been discontinued during various phases of the trial.

### Cytisine Derivatives

Cytisine has been used extensively as a template for nAChR ligands development (Cassels et al., 2005). In addition to varenicline, Pfizer has developed other cytisine derivatives which are nAChR partial agonists (CP-360288, CP-601927 and CP-601932) as part of its drug discovery programs targeting smoking cessation (Coe et al., 2005b). CP-601927 is a high affinity, selective partial agonist at the α4β2 nAChR. In contrast, CP-601932 has similar affinity for α4β2 and α3β4 nAChRs with very low efficacy (2%) at α4β2 nAChRs rendering it as functional antagonist of α4β2 nAChRs (Chatterjee et al., 2011; Mineur et al., 2011). CP-601932 and CP-601927 were safe in human clinical studies but CP-601932 was discontinued in Phase 2 due to a lack of efficacy compared with varenicline. The cytisine dimer, 1,2-bis-N-cytisinylethane (CC4) also acts as partial agonist with apparent selectivity for α4β2 and α6β2 nAChRs (Sala et al., 2013). In a zebrafish model, CC4 and CC26 reduced nicotine-induced self-administration and conditioned place preference (Ponzoni et al., 2014). Dianicline (SSR591813) is another cytisine derivative that was developed by Sanofi-Synthelabo Research, France as a partial agonist with higher affinity for α4β2 nAChRs than other nAChR subtypes. Dianicline increased extracellular dopamine levels in the NAc, and prevented nicotine withdrawal signs in rats (Cohen et al., 2003). Dianicline was well tolerated and reduced self-reported tobacco craving and withdrawal symptoms. However, it did not increase cigarette smoking abstinence rates and was therefore discontinued in Phase 3 due to a lack of efficacy (end of treatment odds ratio of 1.2; Rollema et al., 2010; Tonstad et al., 2011).

### ABT-418 and ABT-089

For more than two decades, Abbott has been investing heavily in the development of safe and effective ligands to target neuronal nAChRs for the treatment of a variety of neuropathologies and for smoking cessation (Arneric et al., 2007). This led to the introduction of a series of nAChR agonists and partial agonists including ABT-418 and ABT-089. ABT-418 is a full nAChR agonist with apparent selectivity for the neuronal nAChR especially the α4β2 subtype (Decker et al., 1994). ABT-418 is 3–10 fold more potent than nicotine in enhancing learning and memory. It has anxiolytic-like effects, a lower potency than nicotine in enhancing dopamine release, and much less propensity to cause side effects associated with nicotine (Arneric et al., 1994; Papke et al., 1997). ABT-418 was well tolerated and had no signs of abuse potential (Potter et al., 1999; Wilens et al., 1999) but its feasibility as smoking cessation aid has not been demonstrated in large scale clinical trials. ABT-089 has higher affinity for the α4β2 than the α7 nAChR subtype, enhanced ACh release and cognitive performance but was less potent and less efficacious than nicotine in stimulating dopamine release (Sullivan et al., 1997). In animal studies, acute administration of ABT-089 had no effect on nicotine self-administration but attenuated the reinstatement of nicotine-seeking behavior (Lee et al., 2014). While ABT-089 was safe and well tolerated in humans; its clinical efficacy as smoking cessation aid has not been yet demonstrated (Wilens et al., 2006; Apostol et al., 2012).

### Mecamylamine and other nAChR Antagonists

Since the beneficial effect of NRTs could be attributed to functional antagonism, due to nAChR desensitization with continuous exposure to agonist, nAChR antagonists were exploited as smoking cessation aids (Dwoskin et al., 2004). Mecamylamine is a non-selective nAChRs competitive antagonist that inhibits nicotine-induced dopamine release from striatal slices in a concentration-dependent manner (Nickell et al., 2013). However, mecamylamine used alone or in combination with nicotine was no better than NRTs used alone in improving the chance of quitting smoking in a double-blind randomized clinical trial (Glover et al., 2007). In recent preclinical studies, selective α6-containing nAChR antagonists such as α-conotoxin PIA and BPiDI have been shown to reduce nicotine-induced DA release in striatal slices and to decrease nicotine self-administration in rats (Brunzell et al., 2010; Gotti et al., 2010; Wooters et al., 2011). These results provided evidence that selective inhibition of α6-containing nAChRs has potential therapeutic use for smoking cessation (Brunzell et al., 2014).

### Sazetidine A (a nAChR Silent Desensitizer)

Sazetidine A represents a new class of nAChR ligands. It does not directly activate nAChR, does not inhibit nicotine-induced nAChR activities and does not induce nAChR up-regulation. Instead, It desensitizes nAChR for prolonged periods without activation (“Silent desensitizer”; Xiao et al., 2006; Zwart et al., 2008; Hussmann et al., 2012). Sazetidine A binds with higher affinity at α4β2 than other nAChR subtypes and shows reduced withdrawal symptoms and nicotine self-administration (Levin et al., 2010), albeit with different functional and behavioral profiles than varenicline (Turner et al., 2013).

## The Potential Therapeutic Use of nAChR Allosteric Modulators

Selective targeting of nAChRs is a leading strategy for smoking cessation aid development, and there are sincere efforts to develop nAChR ligands with high selectivity for specific receptor subtypes. Nevertheless, nicotine and varenicline are the only FDA-approved nAChR ligands for smoking cessation (Hurst et al., 2013). The development of nAChR based therapeutics remains challenging, in large part, because of the presence of multiple neuronal nAChR subtypes in the reward pathway with unique roles in the development of nicotine addiction. The majority of currently pursued nAChRs therapeutics fall under the category of agonists, partial agonists or antagonists that bind to orthosteric (agonist binding) sites. Because neuronal nAChRs subtypes share conserved ACh binding sites, there have been difficulties developing clinically useful agonist/partial agonist with selectivity for a nAChR subtype. Furthermore, direct activation of neuronal nAChRs by orthosteric ligands is associated with alteration in cholinergic transmission due to prolonged activation and desensitization of nAChRs (Williams et al., 2011). Therefore, positive allosteric modulators (PAMs) of nAChRs were introduced as a novel class for nAChR-based therapeutics (Taly et al., 2009). PAMs do not bind to the ACh binding site or activate nAChRs in the absence of ACh. Rather, they potentiate ACh-induced response by increasing ACh potency, enhancing ACh efficacy and/or nAChR opening probability and have minimal effect on the patterns of brain cholinergic activities (Williams et al., 2011; Uteshev, 2014). As such, nAChR PAMs lack reinforcing actions of their own but replace the subjective reinforcement effect of nicotine. Thus, they reduce the need for tobacco intake with minimal abuse liability (Liu, 2013). Since PAMs binding site(s) are distinct from the evolutionarily conserved ACh binding sites, they exhibit greater structural diversity and are more likely to be exclusive to a nAChR subpopulation. As such, nAChR PAMs may provide the required specificity for developing novel compounds that target nAChR subtypes with complex subunit composition (α4α6β2β3, α6β2β3 and α6β2) that are expressed mainly in striatal dopaminergic neurons (Taly et al., 2009). In support of this notion, several available nAChR PAMs have displayed far greater nAChR subtype selectivity than agonists (Sala et al., 2005; Albrecht et al., 2008; Springer et al., 2008; Timmermann et al., 2012; Olsen et al., 2014). Examples of non-selective nAChR PAMs include physostigmine and galantha-mine and selective α4β2 nAChR PAMs include dFBr (desformylflustrabromine), NS9283 (3-[3-(pyridin-3-yl)-1,2,4-oxadiazol-5-yl]benzonitrile, and CMPI (3-(2-chlorophenyl)-5-(5-methyl-1-(piperidin-4-yl)-1H-pyrrazol-4-yl)isoxazole; Figure 2). Galanthamine and physostigmine are used clinically as acetylcholinesterase inhibitors. They were among the first nAChRs allosteric potentiators to be identified (Maelicke et al., 2001). Galanthamine potentiates ACh-induced activities of nAChRs but not muscarinic acetylcholine receptors (Samochocki et al., 2003). Galanthamine has been shown to reduce nicotine-self administration behavior in rats (Liu, 2013) and to enhance dopaminergic neurotransmission *in vivo* via nAChR potentiation (Schilström et al., 2007). The lack of nAChR subtype selectivity hindered the use of physostigmine and galanthamine for smoking cessation but they are valuable pharmacology tools for studying the interaction of allosteric modulators with nAChRs. Physostigmine and galanthamine computational docking studies and photoaffinity labeling with [^3^H]physostigmine and [^3^H]galanthamine identified allosteric binding sites in the nAChR extracellular domain both at the agonist-binding (“canonical”) and non-agonist binding “non-canonical” subunit interfaces (Luttmann et al., 2009; Hamouda et al., 2013). dFBr was isolated from the marine bryozoan *Flustra* *foliacea* (Peters et al., 2002) and its effect as neuronal nAChR PAM was demonstrated. dFBr enhanced ACh-currents in α4β2 nAChR but not α7 or α3-containing nAChRs (Sala et al., 2005; Kim et al., 2007). dFBr was also shown to block the inhibitory effect of β Amyloid (Aβ_1–42_) peptide on α4β2 nAChR (Pandya and Yakel, 2011). In rats, dFBr was shown to reduce intravenous nicotine self-administration without supporting self-administration behavior (Liu, 2013). CMPI and several other substituted piperidines have been identified as potent and selective α4β2 nAChR potentiators in high-throughput screening and lead optimization projects at Amgen Inc., (Albrecht et al., 2008; Springer et al., 2008). NS9283, developed at Neurosearch Inc., specifically enhances the potency of acetylcholine induced currents in α4β2 receptors with a 3α, 2β subunit stoichiometry (Timmermann et al., 2012; Olsen et al., 2014) thus providing further selectivity for a subpopulation within the α4β2 nAChR subtype.

## Conclusion

Drugs that selectively target a subpopulation of nAChRs within the brain’s nicotine reward pathway will have a great impact on the treatment of nicotine addiction. However, the design and development of novel nAChR orthosteric and allosteric ligands require: (1) an accurate mapping of anatomical distribution and delineation of functional contribution of various nAChRs; (2) precise understanding of the three dimensional structure of individual nAChR subtypes; (3) structural studies to illustrate drugs binding mode within the orthosteric binding site (e.g., Hansen et al., 2008; Bach et al., 2015). Such studies will provide structural information that may expand the chemical space in the development of novel partial agonists as a smoke cessation aids; and (4) structural studies to identify binding sites for nAChR PAMs (e.g., Seo et al., 2009; Olsen et al., 2013; Hamouda et al., 2015) and to understand the structural bases of PAM subtype selectivity. Such studies have the potential to identify novel allosteric sites that are unique to α6β2 and α4β2-containing nAChRs and to facilitate the development of novel nAChR based therapeutics for smoking cessation.

## Author Contributions

Contributed to writing of manuscript: TSM, SSJ and AKH.

## Conflict of Interest Statement

The authors declare that the research was conducted in the absence of any commercial or financial relationships that could be construed as a potential conflict of interest.

## Abbreviations

ACh: acetylcholine
DA: dopamine
GABA_A_R: γ-aminobutyric acid type A receptor
nAChR: nicotinic acetylcholine receptor
NAc: nucleus accumbens
NRTs: nicotine replacement treatments
VTA: ventral tegmental area.
